# Physical activity and quality of life of patients with inflammatory bowel disease

**DOI:** 10.1097/MD.0000000000026290

**Published:** 2021-07-09

**Authors:** Bun Kim, Jisuk Chae, Eun Hye Kim, Hyuk In Yang, Jae Hee Cheon, Tae Il Kim, Won Ho Kim, Justin Y. Jeon, Soo Jung Park

**Affiliations:** a Department of Medicine, The Graduated School, Yonsei University College of Medicine, Seoul; b Center for Colon Cancer, Center for Cancer Prevention and Detection, National Cancer Center, Goyang; c Department of Sport and Leisure Studies, Yonsei University; d Department of Internal Medicine and Institute of Gastroenterology, Yonsei University College of Medicine, Seoul, Republic of Korea.

**Keywords:** health-related quality of life, inflammatory bowel disease, physical activity

## Abstract

This study examined the association between physical activity (PA) and quality of life (QOL) in Korean patients with inflammatory bowel disease (IBD).

We enrolled 158 patients with IBD (81 men and 47 women). PA levels were assessed using the International PA questionnaire. Using self-reported frequency (day) and duration (h) of physical activities, the patients were categorized into 3 groups based on their total metabolic equivalent (MET-h/wk) values: least, moderate, and most active. The QOL of patients with IBD was assessed using the inflammatory bowel disease questionnaire (IBDQ), the Medical Outcomes Study 36-Item Short Form Version 2 (SF36v2), the EuroQOL five dimensions questionnaire (EQ5D), and the EuroQOL visual analog scale (EQ-VAS).

Of 158 patients, 62, 73, and 23 patients with Crohn disease, ulcerative colitis, and intestinal Behçet disease, respectively, were included. The mean age was 45.96 ± 17.58 years, and 97 (61.4%) patients were men. Higher PA levels correlated with higher EQ5D and EQ-VAS scores (*P* < .001 and *P* = .004 respectively). In addition, depending on the type of PA, the amount of leisure activity was associated with higher IBDQ (*κ* = 0.212, *P* = .018), physical function of SF36v2 (*κ* = 0.197, *P* = .026), EQ5D (*κ* = 0.255, *P* = .002), and EQ-VAS (*κ* = 0.276, *P* = .001) scores. The frequency of sweat-inducing exercise showed an inverse correlation with IBDQ (*κ* = –0.228, *P* = .011), physical function of SF36v2 (*κ* = –0.245, *P* = .006), EQ5D (*κ* = –0.225, *P* = .007), and EQ-VAS (*κ* = –0.246, *P* = .004) scores.

Increased PA levels were associated with improved QOL in patients with IBD. More leisure activity and non-sweat-inducing exercise were associated with improved QOL in patients with IBD.

## 1 Introduction

Inflammatory bowel disease (IBD), which includes Crohn disease (CD), ulcerative colitis (UC), and intestinal Behçet disease (BD), is a chronic intestinal disease with repeated episodes of relapse and remission due to unknown causes.^[1–3]^

Gastrointestinal and extra-intestinal discomfort negatively affect the quality of life (QOL) of patients with IBD.^[4–8]^ Besides somatic deterioration, the typical relapsing course of IBD often leads to psychological distress, which evokes further impairment. Thus, patients with IBD are frequently affected by depressive syndromes.^[9–12]^ Patients with IBD generally have higher levels of daily stress and lower QOL compared with healthy subjects and even patients with other chronic diseases.^[13,14]^

IBD has no cure despite multimodal medical treatments, including immune-modulating drugs and anti-tumor necrosis factor antibodies. Drug therapy aims at altering the course of disease, reducing its’ symptoms, and improving health-related QOL (HRQOL).^[15–18]^ Therefore, strategies, including complementary pharmacological approaches and psychosocial support, are commonly used by patients with IBD.^[19–21]^

Regular physical activity (PA) has become an important complementary treatment strategy in several chronic diseases, including coronary heart disease, metabolic syndrome, heart failure, breast cancer, and depression.^[22–25]^ However, PA as a therapeutic option for IBD has not been studied sufficiently. Information on the effects of regular PA on disease activity, inflammation, and QOL is limited.^[26]^ Similar to other chronic diseases, muscle function, peak power, and peak oxygen uptake are reduced in patients with IBD.^[27,28]^ Furthermore, to date, no negative side effects of moderate exercise on the physical condition of patients with IBD have been observed.^[29]^ These data suggest that PA may be a safe method for patients with IBD in order to minimize the side effects of IBD and improve HRQOL.

Therefore, the present study focused on examining the association between PA and QOL in patients with IBD in Korea.

## 2 Materials and methods

### 2.1 Study population

The protocols of the research were approved by the institutional review board of Severance Hospital (4–2014–0364). Self-administered questionnaires that measure PA and HRQOL were used. The protocol was first explained to patients with IBD, and informed consent was provided by the patients who agreed to participate before receiving and answering the questionnaires. The data of 158 patients with IBD (62 with CD, 73 with UC, and 23 with intestinal BD) between March 3, 2013 and December 31, 2013 who submitted questionnaires were included in this study. The questionnaires were first distributed at an IBD workshop that was held at Severance Hospital on March 3, 2013. The questionnaires (n = 111) were submitted to 1 of 8 research staff who were familiar with the questionnaire and had previously been trained to help explain the questionnaires to patients with IBD. Additional questionnaires (n = 47) were distributed by health care providers at Severance Hospital who had previously been briefed on the purpose of this study and had agreed to play an active role in the distribution of the questionnaires.

### 2.2 Measurement of physical activity

The exercise participation of patients with IBD was assessed using the Godin leisure-time exercise questionnaire.^[30]^ The questionnaire has 2 items regarding leisure time physical activity: frequency of mild (minimal effort), moderate (not exhausting), strenuous (heart beats rapidly) exercise of at least 15-minutes or more per week, and the frequency of participation in sweat-inducing activities during a typical week. In addition, the mean duration of each exercise intensity was assessed in order to calculate metabolic equivalent hours per week (MET-h/wk). Patients were then categorized into 3 groups based on their total metabolic equivalent (MET-h/wk) values: least, moderate, and most active.

### 2.3 Measurement of QOL

The QOL of patients with IBD was assessed using the inflammatory bowel disease questionnaire (IBDQ), the Medical Outcomes Study 36-Item Short Form Version 2 (SF-36v2), the EuroQOL 5 dimensions questionnaire (EQ5D), and the EuroQOL visual analog scale (EQ-VAS).

The IBDQ is a validated questionnaire that assesses disease-related dysfunction in patients with IBD. This questionnaire consists of 32 questions covering 4 QOL dimensions (bowel, systemic, social, and emotional). Up to 7 points can be given to each question, 224 being the highest achievable score (range, 32–224), and higher values indicate a better HRQOL.^[31]^

The SF-36v2 (QualityMetric, Lincoln, RI) is a generic instrument for measuring HRQOL. The SF-36v2 uses norm-based scoring wherein the scale and component summary scores have a mean and standard deviation (SD) of 50 and 10, respectively, in the US general population. It consists of 36 questions that measure functional health and well-being from the patient's point of view and has been found to be valid and reliable after extensive psychometric evaluations. Responses to items are computed into an 8-domain profile of the scores: physical functioning, role-physical, bodily pain, general health, vitality, social functioning, role-emotional, and mental health (MH). In addition, SF36v2 physical and MH scores (physical health and MH, respectively) are generated. In this study, the higher values indicate a better HRQOL.^[32]^

The EQ5D is one of the most commonly used generic questionnaires to measure HRQOL. The conceptual basis of the EQ-5D is a holistic view of health, which includes the medical definition, as well as the fundamental importance of independent physical, emotional, and social functioning.^[33]^ The descriptive system is composed of 5 dimensions (mobility, self-care, usual activities, pain/discomfort, and anxiety/depression). Each dimension has 5 levels: no, slight, moderate, severe, and extreme problems. The respondent is asked to indicate his/her health state by checking (or placing a cross) the box against the most appropriate statement in each of the 5 dimensions. The digits for 5 dimensions can be combined in a 5-digit number describing the respondent's health state. The index scores are based on general population valuation surveys that used time trade-off methods in various countries. In this study, we used the Korean value set, and higher values (range, 0–1 point) indicate a better HRQOL.^[34]^

The EQ-VAS derives information regarding the patients’ subjective health perception, scored on a visual analog scale with endpoints labeled “the best health you can imagine” and “the worst health you can imagine.”^[35]^ In this study, the higher values (range, 0–100 points) indicate a better HRQOL.

### 2.4 Questionnaire validation

The questionnaires that were used were forward-translated to Korean, and back-translated separately, by experts in the field and compared afterward. The translated questionnaires were subsequently examined by an expert panel for face validation before being used in this study.

### 2.5 Statistical analysis

All statistical analyses were performed using SPSS 21 (IBM Corp., Armonk, NY). Continuous and categorical data were presented as mean (SD) and frequency (percentage), respectively. For the univariate analyses, in the comparison of the 3 groups by PA, a one-way analysis of variance (ANOVA) followed by a post-hoc Bonferroni test was used to analyze the relationship between the PA level and the HRQOL. The correlation between leisure activity and the HRQOL was analyzed with a κ test. The results were considered statistically significant if *P* < .05.

## 3 Results

### 3.1 Patient characteristics

We enrolled 158 patients between March 3, 2013 and December 31, 2013. Of 158 patients, 62, 73, and 23 patients with CD, UC, and intestinal BD, respectively, were included. The baseline characteristics of 158 patients are described in Table 1. The mean age of the patients was 45.96 ± 17.58 years, and 97 (61.4%) patients were men. Only 2 (1.3%) patients were current smokers and 19 (12.0%) patients drank alcohol more than once a week. Educationally, most patients were (84.5%) high-school graduates or higher. Among 158 patients, 139 (88.0%) patients were in clinical remission at the time of the study. In terms of medical treatment, 151 (95.6%) patients among 158 patients took 5-acetylsalicyclic acid and 58 (36.7%) patients used azathioprine or 6-mercaptopurine at the time of the study. Biologics were prescribed in 30 (19.0%) patients among 158 patients. Among biologics, two-thirds were infliximab and the others were adalimumab.

**Table 1:** Characteristics of patients with inflammatory bowel disease.

	Crohn disease (n = 62)	Ulcerative colitis (n = 73)	Intestinal Behçet disease (n = 23)
Male, n (%)	40 (64.5)	45 (61.6)	12 (52.2)
Age (mean ± SD, y)	33.95 ± 12.46	54.10 ± 16.10	52.48 ± 16.00
BMI (mean ± SD, kg/m^2^)	19.75 ± 2.92	22.2 ± 3.08	21.36 ± 3.03
Smoking status, n (%)			
Ever smokers	2 (3.2)	7 (9.6)	2 (8.7)
Former smokers	13 (21.0)	26 (35.6)	8 (34.8)
Never smoked	42 (67.7)	35 (47.9)	13 (56.5)
Current smokers	2 (3.2)	0 (0.0)	0 (0.0)
Unknown	3 (4.8)	5 (6.8)	
Alcohol intake, n (%)			
Never drinkers	42 (67.7)	31 (42.5)	13 (56.5)
Scarcely drinkers	11 (17.7)	27 (37)	7 (30.4)
More than once a week	7 (11.3)	10 (13.7)	2 (8.7)
Unknown	2 (3.2)	5 (6.8)	1 (4.3)
Education, n (%)			
Middle school graduate or under	4 (6.5)	11 (15.1)	3 (13.0)
High school or college graduate	33 (53.2)	36 (49.3)	12 (52.2)
Higher than college graduate	24 (38.7)	24 (32.9)	6 (26.1)
Unknown	1 (1.6)	2 (2.7)	2 (8.7)
Income, n (%) (10,000 won)			
No income/∼200	15 (24.2)/18 (29.1)	13 (17.8)/14 (19.2)	6 (26.1)/8 (34.7)
201–400/401–1000	13 (21.0)/9 (14.5)	16 (21.9)/18 (24.7)	4 (17.4)/2 (8.7)
>1001/Unknown	1 (1.6)/6 (9.7)	5 (6.8)/7 (9.6)	2 (8.7)/1 (4.3)
Marital status, n (%)			
Single/married	36 (58.1)/25 (40.3)	14 (19.2)/50 (68.5)	3 (13.0)/18 (78.3)
Separated/divorced/unknown	0 (0.0)/ 1 (1.6)/0 (0.0)	4 (5.5)/1 (1.4)/4 (5.5)	1 (4.3)/1 (4.3)/0 (0.0)
Disease status, n (%)			
Clinical remission	50 (80.6)	69 (94.5)	20 (87.0)
Mild	12 (19.4)	4 (5.5)	2 (8.7)
Moderate–severe	0	0	1 (4.3)
Medication (currently in use), n (%)			
5-ASA	57 (91.9)	71 (97.3)	23 (100)
Azathioprine and 6-MP	34 (54.8)	15 (20.5)	9 (39.1)
Infliximab/Adalimumab	15 (24.2)/8 (12.9)	4 (5.5)/2 (2.7)	1 (4.3)/0
MTX	3 (4.8)	0	0
Steroid	2 (3.2)	2 (2.7)	3 (13.0)

5-ASA = 5-acetylsalicyclic acid, 6-MP = 6-mercaptopurine, BMI = body mass index, MTX = methotrexate, SD = standard deviation.

### 3.2 Physical activity participation based on the type of IBD

Exercise participation of patients based on the type of IBD can be seen in Table 2. Of 158 patients, 138 (59 patients with CD, 61 patients with UC, and 18 patients with intestinal BD) answered the questionnaire regarding PA. The mean duration of exercise in patients with IBD was 103 min/wk (7.04 ± 10.93 MET-h/wk), with patients with intestinal BD being the least active (5.63 MET-h/wk), and UC, being the most active (7.81 MET-h/wk). Patients with intestinal BD did not participate in any strenuous exercise. However, mild and moderate physical activities were similar between patients with intestinal BD or CD.

**Table 2:** Physical activity participation of patients with inflammatory bowel disease.

	Crohn disease (n = 59)		Ulcerative colitis (n = 61)		Intestinal Bechet disease (n = 18)	
Physical activity	(min/wk)	(MET-h/wk)	(min/wk)	(MET-h/wk)	(min/wk)	(MET-h/wk)
Mild	58.07 ± 105.55	3.00 ± 5.34	66.57 ± 100.50	3.32 ± 5.07	56.67 ± 77.61	2.98 ± 3.93
Moderate	29.92 ± 62.49	2.53 ± 5.24	35.50 ± 77.34	3.19 ± 6.64	33.57 ± 79.42	2.94 ± 6.76
Strenuous	8.31 ± 32.81	1.25 ± 4.92	10.14 ± 39.80	1.59 ± 6.10	0	0
Total	96.29 ± 155.12	6.64 ± 11.85	112.21 ± 132.68	7.81 ± 11.02	90.24 ± 108.15	5.63 ± 7.50

Physical activity measured by Godin leisure-time exercise questionnaire. Values are presented as mean ± SD. MET = metabolic equivalent task.

### 3.3 Relationship between the PA level and HRQOL

All patients were divided into 3 groups based on their PA, and the mean ± standard deviation of the questionnaires is presented in Table 3, which clearly shows the relationship between PA and QOL. The IBDQ scores were higher in the most active group than in the least active group (170.37 ± 42.67 vs 147.35 ± 40.63; *P* = .026). In the SF-36v2, physical functioning and the SF-36v2_PH domains scored higher in the most active group than in the least active group (physical functioning: 23.94 ± 3.63 vs 21.24 ± 5.28; *P* = .018, SF-36v2_PH: 60.67 ± 4.59 vs 57.33 ± 8.31; *P* = .049). The same trend could also be seen for the EQ5D and the EQ-VAS scores (EQ5D: 0.87 ± 0.14 vs 0.70 ± 0.31; *P* < .001, EQ-VAS: 74.29 ± 17.74 vs 61.54 ± 21.12; *P* = .004).

**Table 3:** Relationship between the level of physical activity and health-related quality of life.

	Godin leisure-time exercise questionnaire rate tertile						
	T1		T2		T3		
Variables	n	Total points	n	Total points	n	Total points	*P*-value
IBDQ	40	147.35 ± 40.63	40	162.90 ± 33.16	46	170.37 ± 42.67^∗^	.026
SF36v2	42		43		46		
Physical functioning		21.24 ± 5.28		22.61 ± 4.12		23.94 ± 3.63^∗^	.018
Role-physical		14.62 ± 4.98		15.49 ± 4.66		16.67 ± 4.16	.113
Body pain		4.55 ± 2.00		4.30 ± 2.12		4.02 ± 2.12	.498
General health		16.93 ± 2.10		16.44 ± 2.11		16.04 ± 2.66	.204
SF-36v2_PH		57.33 ± 8.31		58.84 ± 5.67		60.67 ± 4.59^∗^	.049
Vitality		13.62 ± 2.77		13.77 ± 2.00		13.02 ± 2.41	.304
Social functioning		5.60 ± 1.17		5.72 ± 0.91		5.41 ± 1.09	.389
Role-emotional		11.10 ± 4.08		11.47 ± 3.51		12.70 ± 3.20	.094
Mental health		17.74 ± 3.04		17.74 ± 2.44		18.11 ± 2.17	.736
SF-36v2_MH		48.05 ± 8.44		48.70 ± 6.26		49.24 ± 5.59	.717
EQ5D	54	0.70 ± 0.31	46	0.86 ± 0.17^∗^	52	0.87 ± 0.14^∗^	<.001
EQ-VAS	45	61.54 ± 21.12	45	67.33 ± 16.29	51	74.29 ± 17.74^∗^	.004

Mean ± SD in total points for health-related quality of life questionnaires. T1: least active, T2: moderately active, T3: most active. EQ5D = the EuroQOL five dimensions questionnaire, EQ-VAS = the EuroQOL visual analog scale, IBDQ = the Inflammatory Bowel Disease Questionnaire, MH = mental health, PH = physical health, SF-36v2 = the Medical Outcomes Study 36-Item Short Form Version 2.

∗ Significantly different from T1.

### 3.4 Correlation between the type of PA and the HRQOL questionnaires

The Godin leisure-time exercise questionnaire as a PA measurement includes both leisure activity and sweat-inducing exercise. The correlation between the PA type and the HRQOL questionnaires is presented in Table 4. The amount of leisure activity was associated with a higher IBDQ (*κ* = 0.212, *P* = .018), physical function of the SF36v2 (*κ* = 0.197, *P* = .024), the EQ5D (*κ* = 0.255, *P* = .002), and the EQ-VAS (*κ* = 0.276, *P* = .003) scores. In contrast, the frequency of sweat-inducing exercise showed an inverse correlation with the IBDQ (*κ* = –0.228, *P* = .011), physical function of the SF36v2 (*κ* = –0.245, *P* = .026), the EQ5D (*κ* = –0.225, *P* = .007), and the EQ-VAS (*κ* = –0.246, *P* = .004) scores. Sweat-inducing exercise was associated with better general health (*κ* = 0.195, *P* = .028).

**Table 4:** Pearson correlation coefficients between type of physical activity and health-related quality of life questionnaires.

	Godin leisure-time exercise questionnaire							
	Amount of leisure activity				Frequency of sweat-inducing exercise			
Measure of quality of life condition	Model 1		Model 2		Model 1		Model 2	
	*κ*	*P*-value	*κ*	*P*-value	*κ*	*P*-value	*κ*	*P*-value
IBDQ	0.205	.022	0.212	.018	–0.252	.004	–0.228	.011
SF36v2								
Physical function	0.197	.024	0.197	.026	–0.235	.007	–0.245	.006
Role-physical	0.152	.084	0.147	.099	–0.185	.035	–0.159	.075
Body pain	–0.073	.409	–0.060	.505	0.063	.476	0.038	.673
General health	–0.155	.077	–0.161	.071	0.220	.012	0.195	.028
SF-36v2_PH	0.167	.057	0.163	.068	–0.198	.024	–0.198	.026
Vitality	–0.113	.200	–0.127	.154	0.078	.379	0.135	.131
Social functioning	–0.105	.234	–0.099	.267	–0.007	.94	0.003	.974
Role-emotional	0.145	.099	0.144	.107	–0.152	.084	–0.132	.138
Mental health	0.033	.707	0.041	.650	0.007	.941	0.025	.778
SF-36v2_MH	0.034	.703	0.035	.695	–0.054	.542	–0.016	.854
EQ5D	0.258	.002	0.255	.002	–0.242	.003	–0.225	.007
EQ-VAS	0.252	.003	0.276	.001	–0.244	.004	–0.246	.004

Model 1: Crude analysis. Model 2: Age, sex, and body mass index controlled. EQ5D = the EuroQOL five dimensions questionnaire, EQ-VAS = the EuroQOL visual analog scale, IBDQ = the inflammatory bowel disease questionnaire, MH = mental health, PH = physical health, SF-36v2 = the Medical Outcomes Study 36-Item Short Form Version 2.

## 4 Discussion

More recently, PA has been generally regarded as beneficial for patients with IBD as it may counteract some IBD-specific complications by improving the immunological response, psychological health, nutritional status, bone mineral density, and reverse the decrease of muscle mass and strength. Therefore, there have been specific efforts to confirm these favorable effects and to clarify the pathophysiology of PA in IBD.

Most studies have examined the beneficial effects of exercise in terms of QOL and general fitness. Studies on sedentary patients with inactive or mildly active CD have shown that moderate exercise, such as walking or yoga, lead to significant improvements in the measures of the quality of life and levels of stress.^[36–38]^ Those studies also showed that moderate-intensity exercise is well tolerated by patients with IBD who are in remission. Ploeger et al^[39]^ tested the effect of moderate-intensity continuous exercise and high-intensity intermittent exercise in youth with CD. The authors concluded that such patients can engage in distinctly different types of exercise without significant exacerbations of the disease. Although previous research suggested that exercise may be beneficial for patients with IBD, the results of this study suggested that the opposite effect could also be possible regarding the HRQOL. Therefore, further studies are necessary to evaluate the effectiveness of various types of exercise, as well as the duration and intensity of an exercise program to determine an optimal PA level for patients with IBD.

This study has several limitations. First, this study was a cross-sectional study; hence, a causal relationship between PA and HRQOL cannot be determined. Second, the number of patients in each IBD type (CD, UC, and intestinal BD) was small; hence, the relationship between PA and HRQOL in the subgroup of each IBD type could not be analyzed. Finally, because the majority of questionnaires were collected from patients attending an IBD workshop, IBD disease activity at the time of the survey was mostly clinically remission to mild. This could mean that the participants of this study may not accurately represent the general IBD population in Korea. Nevertheless, in patients with IBD, in a state of high disease activity, the measurement of PA or QOL itself might be meaningless, and intensive treatment is usually performed in hospitalization. Therefore, as the subjects of this study, dealing with PA and QOL, it is considered appropriate to target the patients with well controlled disease.

This study is the first study to examine the association of PA and QOL in patients with IBD in Korea, and to the best of our knowledge, in East Asia. The results of this study clearly show that increased PA levels are associated with better QOL in patients with IBD. Moreover, the results of this study also suggest that the impact of PA on QOL may be determined by the type, and, possibly, the intensity, of PA. More specifically, more leisure activities were associated with better QOL; however, sweat-inducing exercise had an inverse association with QOL. This result warrants more research. These findings also demonstrate a need for the establishment of appropriate exercise regime for patients with IBD.

In conclusion, patients with IBD generally have higher levels of daily stress and lower QOL when compared with healthy persons. Regular PA has become an important complementary treatment strategy in several chronic diseases, including coronary heart disease, metabolic syndrome, heart failure, breast cancer, and depression. However, PA as a therapeutic option for IBD has not been studied sufficiently. Particularly, there has been no study regarding the effects of PA on IBD patients in East Asia. Our results illustrate that increased levels of PA are associated with a better quality of life in patients with IBD, even though a causal relationship between PA and health related QOL cannot be determined. Moreover, the results of this study also suggest that the impact of PA on QOL may be determined by the type, and possibly, the intensity, of PA. In this study, more leisure activities were associated with a better QOL; however, sweat-inducing exercise had an inverse association with QOL. Therefore, more research is essential in order to establish the appropriate exercise regime for the improvement of QOL in patients with IBD.

## Author contributions

**Conceptualization:** Jae Hee Cheon, Tae Il Kim, Won Ho Kim, Soo Jung Park.

**Data curation:** Bun Kim, Eun Hye Kim, Soo Jung Park.

**Formal analysis:** Bun Kim, Jisuk Chae, Hyuk In Yang.

**Investigation:** Justin Y. Jeon, Soo Jung Park.

**Methodology:** Jisuk Chae, Hyuk In Yang, Justin Y. Jeon.

**Project administration:** Justin Y. Jeon, Soo Jung Park.

**Supervision:** Jae Hee Cheon, Tae Il Kim, Won Ho Kim, Justin Y. Jeon, Soo Jung Park.

**Writing – original draft:** Bun Kim, Eun Hye Kim.
